# Neurosurgery outcomes and complications in a monocentric 7-year patient registry

**DOI:** 10.1016/j.bas.2022.100860

**Published:** 2022-01-19

**Authors:** Johannes Sarnthein, Victor E. Staartjes, Luca Regli, for the Neurosurgery-Registry consortium, Kevin Akeret, Delal Bektas, David Bellut, Oliver Bichsel, Oliver Bozinov, Elisa Colombo, Sandra Dias, Giuseppe Esposito, Menno R. Germans, Anna-Sophie Hofer, Michael Hugelshofer, Arian Karbe, Niklaus Krayenbühl, Alexander Küffer, Marian C. Neidert, Markus F. Oertel, Luis Padevit, Luca Regli, Jonas Rohr, Ahmed Samma, Johannes Sarnthein, Martina Sebök, Carlo Serra, Victor Staartjes, Lennart Stieglitz, Martin N. Stienen, Lazar Tosic, Tristan van Doormaal, Bas van Niftrik, Flavio Vasella, Stefanos Voglis, Fabio von Faber-Castell

**Affiliations:** b Department of Neurosurgery and Clinical Neuroscience Center, University Hospital Zurich and University of Zurich, Switzerland; a Department of Neurosurgery and Clinical Neuroscience Center, University Hospital Zurich and University of Zurich, Switzerland

**Keywords:** Adverse events, Morbidity and mortality rounds, Quality monitoring

## Abstract

**Introduction:**

Capturing adverse events reliably is paramount for clinical practice and research alike. In the era of “big data”, prospective registries form the basis of clinical research and quality improvement.

**Research question:**

To present results of long-term implementation of a prospective patient registry, and evaluate the validity of the Clavien-Dindo grade (CDG) to classify complications in neurosurgery.

**Materials and methods:**

A prospective registry for cranial and spinal neurosurgical procedures was implemented in 2013. The CDG – a complication grading focused on need for unplanned therapeutic intervention – was used to grade complications. We assess construct validity of the CDG.

**Results:**

Data acquisition integrated into our hospital workflow permitted to include all eligible patients into the registry. We have registered 8226 patients that were treated in 11994 surgeries and 32494 consultations up until December 2020. Similarly, we have captured 1245 complications on 6308 patient discharge forms (20%) since full operational status of the registry. The majority of complications (819/6308 ​= ​13%) were treated without invasive treatment (CDG 1 or CDG 2). At discharge, there was a clear correlation of CDG and the Karnofsky Performance Status (KPS, rho ​= ​-0.29, slope -7 KPS percentage points per increment of CDG) and the length of stay (rho ​= ​0.43, slope 3.2 days per increment of CDG).

**Discussion and conclusion:**

Patient registries with high completeness and objective capturing of complications are central to the process of quality improvement. The CDG demonstrates construct validity as a measure of complication classification in a neurosurgical patient population.

## Highlights

- A prospective registry for cranial and spinal neurosurgical procedures was implemented in 2013.
- We have registered 8226 patients that were treated in 11994 surgeries and 32494 consultations up until December 2020.
- There was a clear correlation of CDG with the Karnofsky Performance Status and with length of hospital stay.
- The Clavien-Dindo grading (CDG) demonstrates construct validity as a measure of complication severity in a neurosurgical patient population.

## Abbreviations

AE: adverse event
CDG: Clavien-Dindo classification grade
CI: 95% confidence interval
CRF: case report form
IQR: interquartile range
KIS: hospital electronic patient record
KPS: Karnofsky Performance Status Scale
LoS: Length of hospital stay
PDCA: Plan-Do-Check-Act
SDH: subdural hematoma

## 1 Introduction

The frequency of postoperative complications is frequently used as an indicator of surgical quality (Theodosopoulos and Ringer, 2015). However, comparison of outcomes is hampered by a lack of agreement on the definition of complications and their classification (Drake et al., 2012; Ferroli et al., 2014). A standard grading system for surgical complications is the Clavien-Dindo classification grading (CDG), which classifies complications according to their need for interventions, and has been validated for the neurosurgical patient population (Sarnthein et al., 2016; Dindo et al., 2004).

Particularly what concerns complications, it has been demonstrated that retrospective data collection systematically underestimates complication rates compared to prospective capture (Campbell et al., 2010). Reliable capture of complications is necessary to monitor trends – e.g. infection rates – as well as to identify systematic human errors, set benchmarks for surgical quality among individual centers and surgeons, and assess the efficacy of new interventions, checklists, and protocols (Rock et al., 2018). Identified problems can then efficiently be targeted in morbidity and mortality conferences or through introduction of quality improvement measures (Rotman et al., 2018).

Stringent prospective surveillance of outcomes and complications is not only paramount for quality improvement, but also forms a basis for answering research questions accurately. Particularly in today's era of “big data”, the large sample size of high-quality, prospective data that can be obtained by running a patient registry for years is highly valuable, enabling powerful statistical and machine learning analyses (Obermeyer and Emanuel, 2016).

We have implemented a neurosurgery patient registry starting in 2013, incorporating the CDG for standardized capture of complications. The patient registry (Sarnthein et al., 2016) has provided data for several further investigations in subgroups of patients (Bellut et al., 2017; Dinevski et al., 2017; Dias et al., 2018; Maldaner et al., 2018a, 2018b, 2019; Schenker et al., 2018; Stienen et al., 2018, 2019; Vasella et al., 2018, 2019; Bucher et al., 2019; Esposito et al., 2019; Henzi et al., 2019; Padevit et al., 2019; Van Niftrik et al., 2019; Zattra et al., 2019; Rautalin et al., 2020; Serra et al., 2020; Staartjes et al., 2020a, 2020b; Voglis et al., 2020; Weber et al., 2022; Terrapon et al., 2021; Yang et al., 2021; Sebök et al., 2021; Jenkins et al., 2021). Complication surveillance using the registry and CDG has enabled targeted quality improvement strategies at our department, too. Furthermore, sharing database methodology and validating outcome measures and complication grading systems such as the CDG for further use by other research groups has proven valuable (Ferroli and Broggi, 2017; Dammann et al., 2020; La Corte et al., 2020; Rybkin et al., 2020; Foster et al., 2021; Vemula et al., 2021). We present here the results of the long-term implementation of our prospective patient registry.

## 2 Methods

### 2.1 Context

The patient registry was designed to fulfil three main purposes, the most important of which is quality monitoring as it is used for communication within the clinic, with external partners, and with patients. Second, the administrative staff uses the registry to monitor the completeness of the patient records. Third, the registry provides an overview over the data available for research projects. The combination of these purposes ensures timely, complete, and accurate data registration.

### 2.2 Intervention

The intervention described here is defined as implementing and maintaining a patient registry and reporting to the department. The registry records data of all patients, which were operated on by members of our neurosurgery department since 2013. Data acquisition to the registry is open-ended. We present here data from the interval 2013–2020.

Typically, a patient is first entered into the registry by the secretary of the surgery theatre. The patient number is scanned from the hospital electronic patient record (KIS). After surgery, the surgeon files the electronic surgery report with a surgery case report form (sCRF) in KIS, which marks the indication and the intervention in the respective catalogue as well as the anatomical localization. The indication catalogue contains one item to state whether the surgery was necessary because of a complication. The surgeon also marks whether the patient was operated on for the first time and whether the surgery had to be rescheduled for medical or administrative reasons.

After surgery, the patient is transferred to the intensive care unit (ICU) and from there to the ward. The resident at the ward files the electronic reports in KIS with one case report form for admission (aCRF) and one for discharge (dCRF). Children ≤16 ​y are transferred to the children's hospital, registered there, and were excluded from the analysis presented here. Patients undergoing a functional surgery (e.g. DBS implantation or epilepsy surgery) are transferred to the neurology department and patients with a vestibular schwannoma are transferred to the otorhinolaryngology department so that there is no dCRF available. At follow-up visits, the surgeon or the physician files an electronic case report form in KIS (fCRF). The CRF are provided as **Supplementary Content**.

### 2.3 Clinical outcome measures

Clinical status at admission is rated on the aCRF in a set of scales comprising the Karnofsky Performance Status Scale (KPS), the modified Rankin Scale (mRS), the Glasgow Coma Scale (GCS), the National Institute of Health Stroke Scale (NIHSS). The aCRF also contains questions on the patients’ social status, their employment and educational level from a set provided by the local health care authorities (Gesundheitsdirektion. Han, 2012). If a hospital admission became necessary because of a complication of a previous treatment, this is recorded on the aCRF in a dedicated matrix.

Clinical status discharge is rated on the dCRF, which contains the same scales as the aCRF. Only the Glasgow Outcome Score (GOS) is applied instead of the GCS. The dCRF also contains a histopathology catalogue of the most common tumor entities. The follow-up case report form (fCRF) contains the same scales as in the dCRF and in addition, also the employment status is marked. The sCRF, aCRF, dCRF and fCRF are exported from KIS and entered into the electronic patient registry.

If a complication occurred, it is marked on the CRF in a catalogue of the most frequent adverse events (AE). Any deviation from the pre-operative status and normal postoperative course is considered a complication: a new motor deficit or a wound infection is counted as a surgical complication; a first time epileptic seizure is also counted as a surgical complication; reoccurring seizures are counted as medical complications caused by inadequate drug dosage; similarly a urinary tract infection. The complication is then graded using the therapy-oriented Clavien Dindo classification system (CDG, Table 1). (Dindo et al., 2004; Clavien et al., 1992; Clavien, 2013) The physician also enters the date of occurrence of the complication and/or the interval in relation to the surgery date. This prevents multiple counting of the same event and also classifies the complication as a transient condition or as a permanent deficit. A deficit is defined as “permanent”, if it persists at the time of the follow-up visit and marked with “d”. In particular, this pertains to new neurological deficits. If the deficit has ceased at the next follow-up visit, its status is changed to “transient”. In case of multiple complications after one surgery, all complications are listed and graded according to CDG (Table 2). For ease of handling, only the complication with the highest CDG is used to characterize that surgery in our analysis of the complication rate.

**Table 1:** Classification of surgical complications.

Grade	Definition
CDG1	Any deviation from the normal postoperative course without the need for pharmacological treatment or surgical, endoscopic, and radiological interventions. Allowed therapeutic regimens are drugs such as antiemetics, antipyretics, analgetics, diuretics, electrolytes, and physiotherapy. This grade also includes wound infections opened at the bedside.
CDG2	Requiring pharmacological treatment with drugs other than those allowed for grade I complications. Blood transfusions and total parenteral nutrition are also included in this grade.
CDG3	Requiring surgical, endoscopic, or radiological intervention.
a	Intervention not under general anesthesia.
b	Intervention under general anesthesia.
CDG4	Life-threatening complication (including CNS complications) requiring IC/ICU management.∗
a	Single-organ dysfunction (including dialysis).
b	Multiorgan dysfunction.
CDG5	Death of a patient.

CNS central nervous system; IC intermediate care; ICU intensive care unit.

∗ Brain hemorrhage, ischemic stroke, and subarachnoidal bleeding but excluding transient ischemic attacks.

**Table 2:**
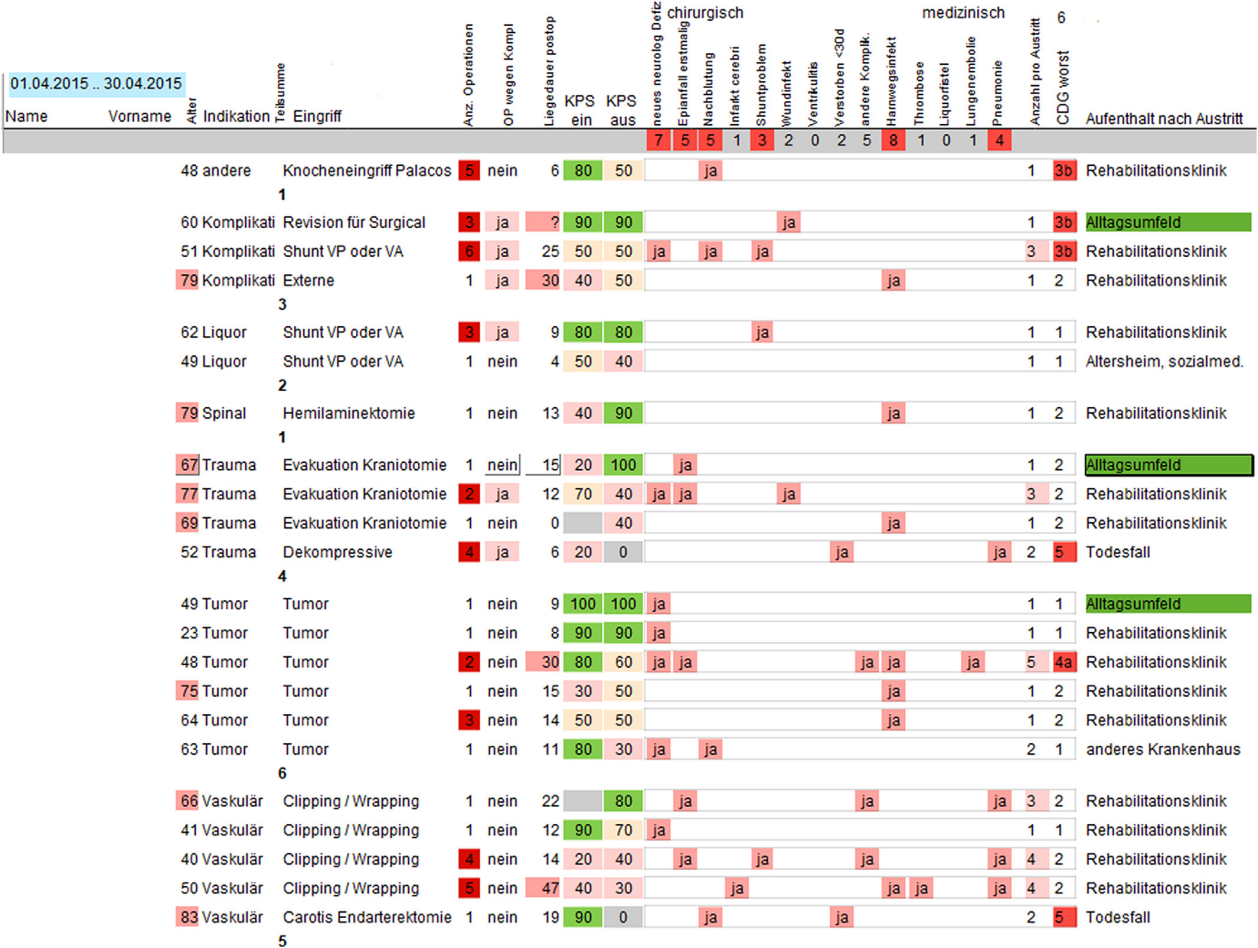
Template for presenting all patients with complications.

### 2.4 Assuring data quality

The resources required exclusively for maintaining the patient registry consist of the secretary of the surgery theatre (1 ​h per day) and the registry manager (JS). The surgeons, the secretary of the surgery theatre and the physicians on the ward need less than 5 ​min to fill out one CRF and a little more time, if information has to be acquired from the electronic patient records. The workload of the other administrative staff has not been changed by the registry, because other data bases were replaced.

The electronic patient registry was implemented in a relational database in filemaker® (www.filemaker.com). The database was custom designed and programmed by the data manager (JS) with external support by www.hyperdots.ch. Several templates and scripts aid in controlling completeness of the data acquisition. Data safety is assured by a hospital file server and user profiles with different levels of authorization.

To assure the completeness of inclusion of patients in the registry, we rely on the administrative staff that supervises the completeness of the patient reports to the external partners. The administrative staff uses the same filemaker® database. Sending out the reports is mandatory for the neurosurgery department to be remunerated. The administrative staff sets a flag in the electronic patient registry as soon as a surgery report is signed by the surgeon and a discharge report is signed by the treating physician of the ward. In this way the state of the patient flow through the clinic is documented for each patient. If a CRF is missing, this is communicated to the resident in charge on the ward. The combined implementation of administrative and clinical in the filemaker® database ensures complete inclusion of all patients in the patient registry.

**Fig. 1:**
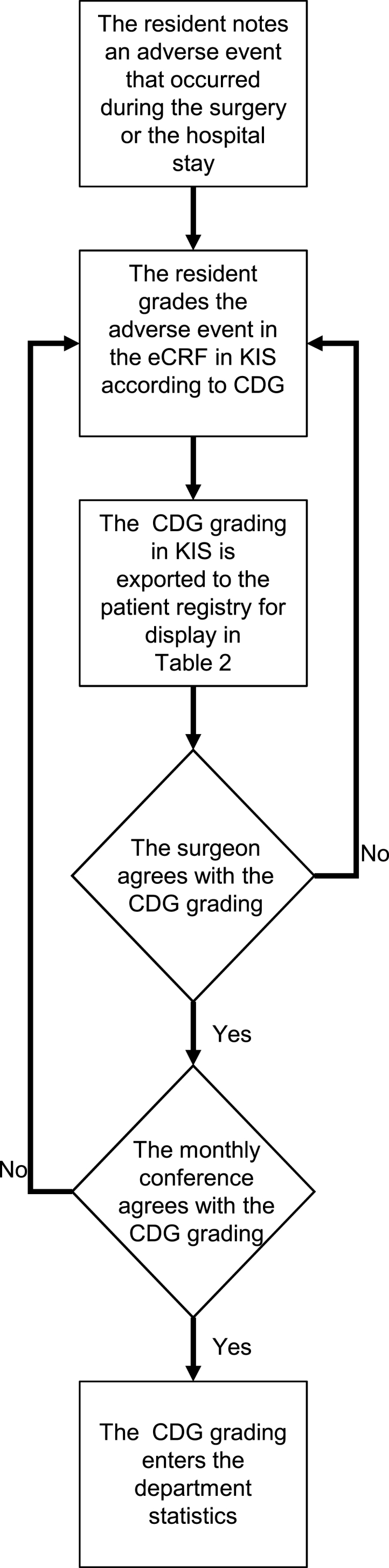
Flowchart of documenting a complication in the patient registry. The diagram is shows how a complication is described in the case report form at discharge (dCRF) and who the complication is classified in a Clavier-Dindo grade (CDG).

To assure the accuracy of the data, each resident undergoes training in the relevant scales before entering data in the CRF. Each CRF that was completed by the resident on the ward is then controlled by the responsible senior physician (Fig. 1). If a correction turns out to be necessary, also the CRF in the electronic patient registry is corrected. All patients with complications are listed on a data analysis template in the electronic patient registry (Table 2), which is regularly discussed in the neurosurgery department at the monthly staff meeting and the monthly morbidity and mortality conference. Here the surgeons and other senior physicians provide the context for the complication. Some surgeries trigger controversial discussions regarding the classification of the complication. In particular, the distinction between sequelae and complications is discussed lively. If necessary, data entries are corrected (Fig. 1).

In research projects on some selected patient groups that are prepared for publication (Table 3), further clinical information is extracted manually from KIS and entered in the CRF of the electronic patient registry. In the registry the data remains readily available for the department also after completion of the research projects.

**Table 3:** Publications that present postoperative CDG.

Publication	N surgeries	Any AE (CDG ≥1)	AE requiring surgery (CDG 3 or 4)	CDG-KPS Spearman's rho	CDG-LoS Spearman's rho
**This study**: All surgeries 2013–2020	11′448	20% CI [19% 21%]	5% CI [5% 6%]	-0.29	0.43
Surgeries with dCRF 2013–2019, Terrapon et al., 2021	4′680	22% CI [20% 23%]	5% CI [ 4% 6%]		
Meningiomas, Jenkins et al., 2021	345	21%			
Unruptured intracranial aneurysms, Sebök et al., 2021	157	13% CI [8% 20%]	3% CI [1% 6%]		0.23
Unruptured intracranial aneurysms, Staartjes et al., 2020b	156	13% CI [ 8% 19%]			
Chronic SDH, Bucher et al., 2019	435	38% CI [34% 43%]	17% CI [13% 21%]	-0.27	0.209
Smokers, Padevit et al., 2019	798	30% CI [23% 37%]			
Surgical Site Infections, Stienen et al., 2019	5′462		1%		
Octogenarians, Maldaner et al., 2018a	266	36% CI [30% 42%]	7% CI [ 4% 10%]	-0.27	0.30
Chronic SDH, Maldaner et al., 2019	253	22% CI [17% 27%]	7% CI [ 4% 11%]		
Shunts, Schenker et al., 2018	195	58% CI [51% 65%]	25% CI [19% 32%]	-0.48	0.46
Lumbar spine, Bellut et al., 2017	138	32% CI [24% 40%]	7% CI [4% 13%]	-0.33	0.4
All surgeries 2013–2015, Sarnthein et al., 2016	3′959	24% CI [22% 27%]	7% CI [6% 9%]	-0.3	0.4

### 2.5 Statistical analysis

To describe variation within the data, we present medians with the interquartile range (IQR) and percentages together with 95% confidence intervals (CI) based on the binomial distribution. We used non-parametric statistical methods for hypothesis testing. The analysis was performed with custom scripts in MATLAB® (www.mathworks.com). Statistical significance was established at p ​< ​0.05. To reduce variation within the data concerning specific aspects of the patient registry, we selected time intervals from the time on when the data acquisition of the specific aspect was fully operational.

### 2.6 Ethical considerations

The scientific workup was approved upfront by the local ethics review board (Kantonale Ethikkommission Zürich PB-2017-00093) and it was registered internationally at clinicaltrials.gov (NCT01628406). The authors report no relevant conflicts of interest. This study is reported in accordance with the STROBE (Strengthening the Reporting of Observational Studies in Epidemiology) statement (Von Elm et al., 2007).

## 3 Results

### 3.1 Completion of CRF over time

**Fig. 2:**
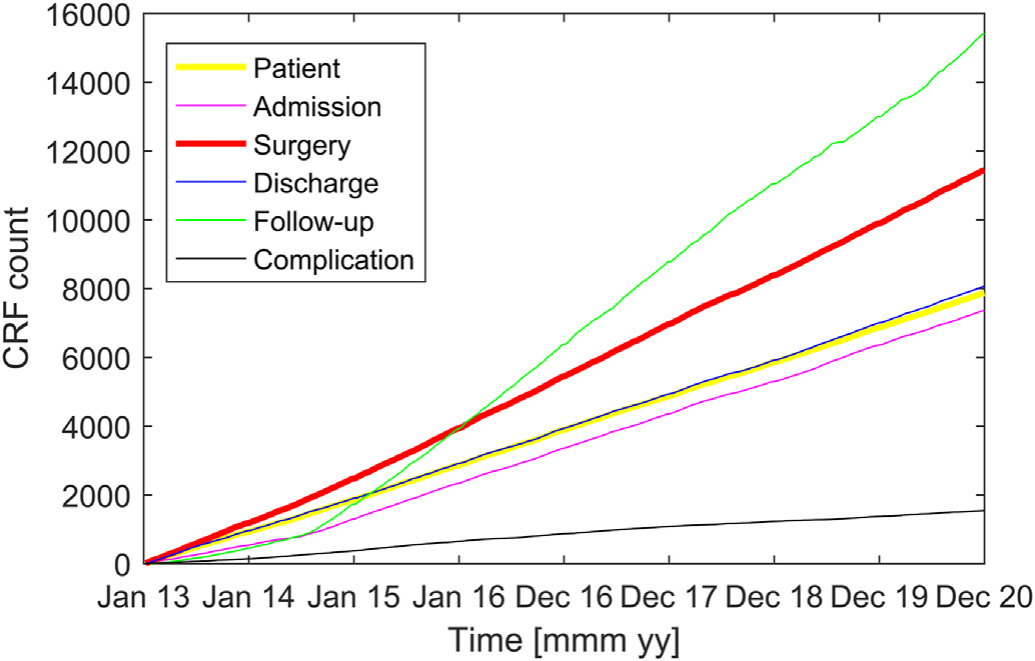
Cumulative sum of case report forms (CRF) Case report forms (CRF) for patients, admissions, surgeries, discharges and follow-ups. Complications registered at discharge (black line).

Fig. 2 shows the number of CRF in the patient registry since its inception. The inclusion of surgeries (sCRF) has proceeded at a regular rate starting from 2013. The number of discharges (dCRF) increases also linearly, albeit at a somewhat lower rate. The lower rate reflects the fact that some patients are operated on several times before discharge and not all patients are discharged from the ward of our department. The slope of the follow-up curve exceeds that of the discharge curve, because several patients appear at several follow-ups. Admissions (aCRF) were included starting summer 2014 and increase in parallel with the discharge curve. The small numbers of admissions and follow-ups before the regular registration were entered from the electronic patient records (KIS) for research projects on select patient cohorts. From August 2014 on, all dCRF were completed with at least a KPS score. Until the end of 2020 we have registered 7869 patients that were treated in 11448 surgeries and 30845 consultations.

### 3.2 Patient and surgery characteristics

The median age of patients at discharge was 60 (IQR 25) years. A discharge from the hospital was preceded by one or more surgeries. On the dCRF, the main indications were neuro-oncology (37%), neurovascular disease (19%), spinal neurosurgery (13%), trauma (10%), cerebrospinal fluid disorder (8%), other (7%), and one or more complications as a separate category of surgical indication (6%, CDG≥ 3). The percentage of spinal interventions is relatively small, which underlines the cranial focus of our department. When counting surgeries by sCRF, there were 977/9530 surgeries indicated because of a complication since August 2014 amounting to a reoperation rate of 10% CI [9% 11%]. This includes complications also from interventions in other hospitals or departments.

### 3.3 Complications at discharge

Fig. 2 shows the rate of complications registered in the dCRF (black line). Complications were registered in the dCRF on a regular basis starting only from August 2014. Of the 6308 dCFR, there were 5063 without complications and 1245 with complications marked (20% CI [19% 21%]), where any deviation from the normal clinical course was registered as a complication, be it a surgical or a medical complication. Further complications, like surgical site infections (SSI), were additionally registered after discharge at follow-up visits.

### 3.4 Complications, KPS at discharge and length of hospitalization

**Fig. 3:**
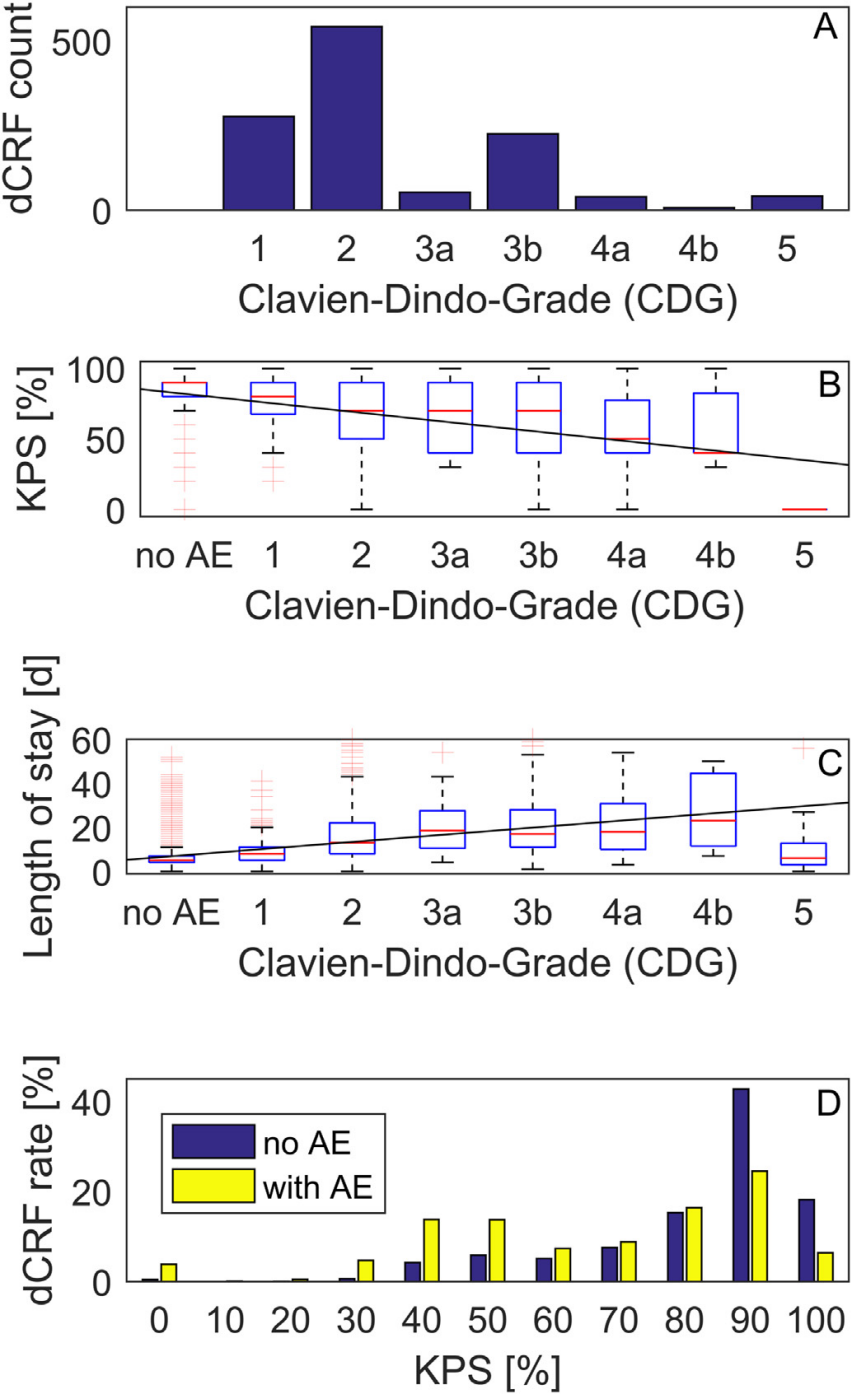
Complications at discharge. (A) Distribution of grades in the Clavien Dindo classification system (CDG). (B) Karnofsky Performance Status Scale (KPS) as a function of grade of the complications (rho ​= ​-0.29, slope -7 KPS percentage points per increment of CDG). (C) Duration of hospital stay after surgery (rho ​= ​0.43, slope 3.2 days per increment of CDG). (D) Distribution of adverse events (AE) across patients with different KPS. The majority of patients with KPS ≥90 (89%) do not show a complication at discharge.

Fig. 3A shows the distribution of the CDG in 1245 complications registered at discharge (dCRF) since August 2014. The majority of complications (819/6308 ​= ​13% CI [12% 14%]) were treated without invasive treatment (CDG 1 and CDG 2). CDG 1 was marked in 277 (4% CI [4% 5%]) patients, including those with a focal neurological deficit, which was not treated and improved in some patients after discharge with the passage of time. Among the most common complications were urinary tract infections with 170/6308 dCRF (3% CI [2% 3%]). One or more additional surgical interventions before discharge (CDG 3 and CDG 4) were listed in 325/6308 (5% CI [5% 6%]) dCRF. The distributions vary between patient cohorts and depend also on the preoperative state of the patient, which is not considered here

The median KPS at discharge was significantly lower for patients with complications (p ​= ​1e-112, Mann-Whitney *U* test). The median KPS for dCRF without complication and dCRF for different CDG is shown in Fig. 3B. The KPS scale and the CDG grade were correlated with Spearman's rho ​= ​-0.29 (p ​= ​4e-120). The linear fit had a slope of -7 KPS percentage points per increment of CDG.

The median postoperative length of hospitalization was significantly higher in cases with a complication (12 vs. 5 days, p ​= ​2e-251, Mann-Whitney *U* test). The length of stay and the CDG grade were correlated with Spearman's rho ​= ​0.43 (p ​= ​1e-287). The linear fit had a slope of 3.2 days per increment of CDG (Fig. 3C).

Fig. 3D shows the distribution of complications among patients with different KPS. The majority of patients with KPS ≥90 do not show any complication at discharge (3084/3468 ​= ​89% CI [88% 90%]). The prevalence of any complication was significantly lower for patients with KPS 90 or higher (p ​= ​2e-81, chi-square-test).

## 4 Discussion

We present our long-term experience with a prospective departmental patient registry, with over 11′448 procedures, 6308 discharges and 1245 complications captured. We describe the registry's methods and surveillance mechanisms, and validate the CDG for neurosurgical patients with sufficient statistical power. Our results demonstrate that setting up a fully functional and complete patient registry is feasible, and that the CDG – as a classification of complications – correlates well with functional outcome and length of hospital stay.

### 4.1 The choice of measures for the registry

The CDG is a therapy-oriented classification of complications, i.e. based on the level of intervention that is required by a certain complication (Dindo et al., 2004). Originating in general surgery, it has been widely applied, including in neurosurgery (Sarnthein et al., 2016). Table 3 reviews publications from our prospective registry that focus on specific patient cohorts. It lists the total percentage of complications (CDG ≥1) at discharge, the percentage of complications that required revision surgery (CDG 3 or CDG 4), and the correlation rho between CDG-KPS and the correlation rho CDG-LoS. These findings, together with the current analysis with much greater statistical power, demonstrate the construct validity of the CDG as a measure of complication classification, according to the COSMIN criteria (Mokkink et al., 2010). Construct validity indicates that a measurement is consistent with the hypothesis that the instrument validly measures the construct to be measured in regard of its relationships to scores of other relevant, validated instruments – in this case KPS as a widely adopted measurement of functional status, and LoS as a driver of immobilization and thromboembolic events, nosocomial infections, and healthcare costs (Bai et al., 2019). In addition, the CDG can easily be cross-walked to the Landriel-Ibanez classification, which constitutes another popular neurosurgical complication classification (Ferroli et al., 2014).

One particular difficulty when setting up a prospective patient registry is choosing a battery of relevant outcome measures. On the one hand, specific outcome measures such as the Oswestry Disability Index for the lumbar spine, the ILAE classification for seizure control after epilepsy surgery, or the House-Brackman scale for facial nerve function are highly valuable when e.g. analyzing outcomes after lumbar spinal fusion, selective amygdalohippocampectomy, or vestibular schwannoma resection. We opted for KPS, modified Rankin scale, and NIHSS as they constitute three widely adopted instruments that can be applied both to patients undergoing cranial as well as spinal procedures.

### 4.2 The registry improves clinical practice

The quality of patient treatment can be monitored in many dimensions. In surgical practice, adverse outcomes such as complications, extended length of stay, reoperations, and unplanned readmissions usually prevail as the dominant driver of patient dissatisfaction, poor quality of life, and healthcare costs (Twitchell et al., 2018; Medress et al., 2020). Demonstrably, these events are poorly captured in retrospective analyses compared to prospective registries (Campbell et al., 2010). To enable effective quality improvement procedures such as morbidity and mortality conferences or protocols and checklists, each department requires accurate surveillance in the form of a patient registry. In addition, systematic capture and scheduled analysis of adverse outcomes identifies systematic errors that may be easily corrected, or to assess the real-world effectiveness of a new intervention at a specific hospital – as opposed to the efficacy described in a clinical trial thereof (Cochrane, 1972). While various quality improvement procedures have proven effective, there is an intense discussion about which benchmarks are to be used to guide such interventions (Theodosopoulos and Ringer, 2015; Drake et al., 2012; Ferroli et al., 2014; Rotman et al., 2018).

The clinical relevance of the patient registry is embedded in a Plan-Do-Check-Act (PDCA) cycle. For example, our institution (University Hospital Zurich) has issued the benchmark to reduce surgical site infections (SSI) to the 5% level (Plan). During patient treatment and surgery, we follow the institutional rules issued by the hospital hygiene department (Do). Monitoring SSI in the patient registry assures a timely monitoring of trends in treatment quality in our monthly morbidity and mortality conferences (Check, Fig. 1, Table 2). If a problem was identified, appropriate quality improvement measures are introduced (Rotman et al., 2018).

Detection rates of common neurosurgical complications have also been demonstrated to vary widely according to local routines for screening, surveillance, documentation, and follow-up (Viken et al., 2018). This demonstrates that quality improvement should not solely be based on complication rates. Rather, outcomes in general, disease severity of the patient population treated at a certain center, as well as readmissions and reoperation rates should also be taken into account. It has even been suggested that reoperations may be a more sensitive surrogate of “quality” in the sense of a surgical quality improvement program (McLaughlin et al., 2015).

On a side note, registries can also be applied to answer more mundane but critically important questions that may vary greatly among each center and can thus not necessarily be answered by considering published literature from other centers in other countries – a prime example is the involvement and degree of involvement of residents in neurosurgical procedures and its relationship with quality of care (Lim et al., 2015). The manner and extent with which neurosurgical residents, particularly in early residency, are involved in procedures can vary greatly by health system and from hospital to hospital. It could thus be argued that a departmental registry is the most reliable way of assessing such aspects, and that for all of the reasons discussed above, monitoring in prospective institutional registries ought to become self-evident for all neurosurgical departments.

### 4.3 Resources required for the registry

It is clear that internal monitoring in the registry requires resources, both in setting up the registry and in running the registry.

Setting up the registry required one senior staff (JS) from the department to manage the design of the registry, to obtain agreement from the ethics committee, to train administrative staff, residents, and attending physicians, to invite and handle change requests from the members of the department, to supervise an external programmer for the database in filemaker®, to interface to the hospital data management (KIS) and to provide the monthly feedback at the internal morbidity and mortality conference. This development took about 5 years.

Running the registry adds to the workload of the department staff. The resident enters the complications and their CDG grading into the eCRF in KIS. The surgeon controls the entries in the eCRF and discusses them with the resident. The registry manager (JS) curates the entries in the patient registry. If there were more items in the eCRF, the resident would be less likely to enter all data entries correctly in the limited time available. Incomplete datasets are very challenging to analyze.

Therefore, a prospective registry can only include a limited number of items to assure the balance between, on one hand, a complete description of the patient's status through many scales and, on the other hand, complete data acquisition through the resident in the clinical routine. The design of our patient registry has evolved to assure this balance given the resources at our disposal in our department.

### 4.4 Limitations and outlook

The completeness of the prospectively recorded data was not always as high as it is now. Compliance with filing the CRFs has increased over the years as the team adjusted to the system. As we transitioned from paper-based to electronic data capturing, the filing of the CRF improved in being more timely and complete. Still, completeness and accuracy of the registry is contingent on the training and motivation of each healthcare practitioner.

For the separate analysis of patient cohorts that we have published up to now (Table 3), missing data was completed retrospectively from the electronic patient records in KIS. While this approach certainly is more complete than no missing data treatment at all or than “last observation carried forward”, some complications may have been missed in these particular patients. Some cohorts consider AE including the 3 months follow-up – for example, surgical site infections are often not apparent at discharge and present as a complication within the first postoperative months. This retrospective addition of data from KIS also included more specialized outcome measurement instruments for specific disease groups. On one hand, from a data analysis perspective, it might be desirable to include more items in the prospective registry. On the other hand, given the limited resources, we have decided for a limited set of items to assure complete data for this set.

It is interesting to stratify patients with respect to baseline differences. In one publication, we have stratified our patient population with respect to their age: neurosurgery in octogenarians had a similar rate of complications, morbidity, and mortality as in matched controls (Maldaner et al., 2018a). Other stratification criteria like KPS at admission, multimorbidity are currently only used for selected surgeries. In future analyses, we aim to stratify our patient population with respect to further baseline conditions.

Patient follow-up can also be achieved automatically using web-based questionnaires sent by e-mail, however incurring a relevantly lower response rate (Pronk et al., 2019). With increasing digitalization of hospitals and patients alike, one could expect applications of machine learning to largely automate data collection for prospective registries. For example, applications of natural language processing (NLP) could record complications in a potentially more objective way (Staartjes and Stienen, 2019). While fully automated data collection using NLP is in theory possible, providing entirely objective and potentially more complete capturing, its introduction is currently still precluded by several barriers.

## 5 Conclusions

Patient registries with high completeness and objective capturing of complications are central to the process of quality improvement, enabling targeted interventions such as morbidity and mortality conferences, introduction of new checklists and protocols, as well as to identify systematic human errors and to monitor the efficacy of newly introduced measures. In this report, we present our long-term experience in setting up and running a prospective departmental patient registry. In addition, we provide evidence for construct validity of the CDG as a measure of complication classification in a neurosurgical patient population.

## Disclosures

None of the authors discloses any perceived Conflict of Interest.

## Declaration of competing interest

The authors declare that they have no known competing financial interests or personal relationships that could have appeared to influence the work reported in this paper.
