# Supplementary material for: Neurosurgery outcomes and complications in a monocentric 7-year patient registry
Source: Brain Spine. 2022 Jan 19;2:100860. doi: 10.1016/j.bas.2022.100860 (PMC9560692; doi:10.1016/j.bas.2022.100860)
Supplement: Multimedia component 1 [file mmc1.pdf]

Supplementary Content: Electronic Case Report Forms

1 Admission Case Report Form (aCRF) 2
2 Surgery Case Report Form (sCRF) 3
2.1 Lokalisation 4
3 Discharge Case Report Form (dCRF) 5
4 Follow-up Case Report Form (fCRF) 7

1 Admission Case Report Form (aCRF)

CRF-Eintritt **Kaderarzt Visum**

Komplikationen

Führten Komplikationen zum Eintritt? ◉ nein

Soziales Umfeld und Ressourcen (Fragen an den Patienten)

Wohnsituation
- ○ Einpersonenhaushalt
- ○ Mehrpersonenhaushalt

Lebt im Haushalt eine Person, die sich um sie kümmert?
- ○ Ja
- ○ Nein

Wie ist Ihre Gesundheit im Allgemeinen?
- ○ Sehr gut
- ○ gut
- ○ mittelmässig
- ○ schlecht
- ○ sehr schlecht

Rauchen Sie?
- ○ ja
- ○ nein, noch nie
- ○ nein, sistiert

Berufliche Situation derzeit
- ○ erwerbstätig 100% (auch wenn arbeitsunfähig / krank)
- ○ erwerbstätig Teilzeit (auch wenn arbeitsunfähig / krank)
- ○ in Ausbildung / Umschulung
- ○ Haushalt / Freiwilligentätigkeit
- ○ keine Tätigkeit
- ○ arbeitslos gemeldet
- ○ IV-Bezüger (Invalidenrente)
- ○ AHV-Bezüger (Altersrente)
- ○ andere
- ○ unbekannt

Ausbildung
- ○ keine abgeschlossene Schulbildung
- ○ obligatorische Volksschule
- ○ Berufslehre / Berufsschule
- ○ Maturitätsschule
- ○ Höhere Fach- oder Berufsschule
- ○ Universität, Hochschule
- ○ unbekannt

Eintrittsstatus

☐ Patient intubiert eingeliefert
(KPS=20; mRS=5; kognitiv=unbekannt)

**Karnofsky-Index**	90 %	29.12.2021
**Glasgow Coma Score**	15	29.12.2021
**Modified Rankin Scale**	1	29.12.2021
**Modifizierte NIH Stroke Scale**	n.d.	29.12.2021

Verhalten ◉ adäquat ○ auffällig ○ unbekannt

Montreal Cognitive Assessment ☒ Nicht erhoben

Epileptischer Anfall präoperativ? ○ ja ◉ nein ○ unbekannt

Neurostatus Neurostatus löschen

Internistischer Status Internistischer Status löschen

2 Surgery Case Report Form (sCRF)

CRF Operationsbericht

Indikation für heutigen Eingriff

Neoplasie

Eingriff

Resektion

Lokalisation

Precentral gyrus

Kontext

Erstoperation: ◉ ja ○ nein

Wurde der Operationstermin verschoben? ◉ nein ○ ja, medizinisch indiziert ○ ja, organisatorisch

Einschätzung Tumor-Resektion

Gliom

○ Complete resection ○ GTR (>98%) ○ Subtotal resection (>90%) ○ Partial resection (<90%) ○ Biopsy

Metastase / anderer Tumor

◉ Totalentfernung ○ Partielle Entfernung ○ Biopsie

Meningeom Simpson ○ 1 ○ 2 ○ 3 ○ 4 ○ 5

ASA Klasse

○ I ◉ II ○ III ○ IV ○ V

2.1 Lokalisation

- ICA
- ACA proximal / ACom
- ACA distal
- MCA proximal
- MCA distal
- Hinterer Kreislauf

- Zentralregion
  - Precentral gyrus
  - Postcentral gyrus
  - Subcentral gyrus
  - Paracentral lobule
- Insula
  - Short insular gyri
  - Long insular gyri
- Hemisphärisch andere
  - Frontal Lobe
    - Superior frontal gyrus
    - Middle frontal gyrus
    - Inferior frontal gyrus
    - Orbital gyri
  - Parietal Lobe
    - Superior parietal lobule
    - Angular gyrus
    - Supramarginal gyrus
    - Precuneus
  - Temporal Lobe
    - Superior temporal gyrus
    - Middle temporal gyrus
    - Inferior temporal gyrus
    - Lateral occipito-temporal gyrus (fusiformis)
    - Medial occipito-temporal gyrus (parahippocampus)
  - Occipital Lobe
    - Superior occipital gyrus
    - Middle occipital gyrus
    - Inferior occipital gyrus
    - Cuneus
    - Lingual gyrus
  - Limbic lobe
    - Subcallosal area
    - Cingulate gyrus
    - Isthmus of the cingulate gyrus
    - Parahippocampal gyrus
    - Uncus
  - White matter of telencephalon
    - Corona radiata
    - Internal capsule
    - Corpus callosum
  - Deep (not basal ganglia or thalamus)
    - Basal forebrain
    - Hypothalamus
- Stammganglien / Thalamus
  - Thalamus
  - Basal ganglia
- Pinealis Region
- Hypophyse
- Hirnstamm
  - Midbrain
  - Pons
  - Medulla oblongata
- Kleinhirn
  - Hemisphere of cerebellum
  - Vermis
- Intraventrikulär
  - Lateral ventricle
  - Third ventricle
  - Fourth ventricle
- Hippocampus / Amygdala
  - Hippocampus
  - Amygdala

- Vordere Schädelgrube (inkl. Nase, Orbita)
- Mittlere Schädelgrube (inkl. Sella, Sinus Cavernosus)
- Hintere Schädelgrube (inkl. Kraniozervikaler Übergang)
- Konvexität / Falx

- Spinal

- Körperstamm

- Sonstige

3 Discharge Case Report Form (dCRF)

CRF Austritt

Kaderarzt Visum visieren | visieren als

Austrittsdaten

Art des Austritts (AQC)
- ○ geheilt (erwartetes Resultat erreicht)
- ○ gebessert
- ○ nicht gebessert / unverändert
- ○ verschlechtert
- ○ nicht beurteilbar
- ○ Exitus intraoperativ
- ○ Exitus postoperativ
- ○ Exitus ohne Zusammenhang mit OP
- ○ Exitus innert 30 Tage nach OP

Aufenthalt nach Austritt (PRISMA)
- ○ Todesfall
- ○ Alltagsumfeld (Privathaushalt o. Ä.)
- ○ Krankenheim, Pflegeheim
- ○ Altersheim, sozialmedizin. Institution
- ○ Psychiatrische Klinik
- ○ Rehabilitationsklinik
- ○ anderes Krankenhaus
- ○ andere
- ○ unbekannt
- ○ eigenes Spital / andere Abteilung

Behandlung nach Austritt (PRISMA)
- ○ Todesfall
- ○ geheilt oder kein Behandlungsbedarf
- ○ ambulante Behandlung
- ○ ambulante Pflege / Spitex
- ○ stationäre Behandlung
- ○ Rehabilitation
- ○ andere
- ○ unbekannt

Exitus Datum

Histopathologie

Neuroepitheliale Tumore
- ☐ Astrocytom Grad II
- ☐ Anaplastisches Astrozytom Grad III
- ☐ Glioblastom
- ☐ Gliosarkom
- ☐ Pilozytisches Astrocytom (WHO Grad I)
- ☐ Pleomorphes Xanthoastrocytom
- ☐ Subependymales Riesenzellastrozytom
- ☐ Oligodendrogliom (WHO Grad II und III)
- ☐ Ependymom
- ☐ Anaplastisches Ependymom
- ☐ Oligoastrozytom
- ☐ Plexuspapillom
- ☐ Gangliocytom
- ☐ Pineocytom
- ☐ Pineoblastom
- ☐ Neuroblastom
- ☐ PNET (incl. Medulloblastom)

Meningeale Tumore
- ☐ Meningeom (WHO Grad I)
- ☐ Atypisches Meningeom (WHO Grad II)
- ☐ Anaplastisches Meningeom (WHO Grad III)

Metastatische Tumore
- ☐ Metastase

Tumore von Hirn- und Spinalnerven
- ☐ Schwannom
- ☐ Neurofibrom
- ☐ Maligner peripherer Nervenscheidentumor

Hämatopoetische Neoplasien
- ☐ Lymphom
- ☐ Plasmozytom

Stammzelltumore
- ☐ Germinom
- ☐ Embryonales Carcinom
- ☐ Coriocarcinom
- ☐ Teratom

Cystische Tumore
- ☐ Rathke'sche Tasche Cyste
- ☐ Epidermoidcyste
- ☐ Dermoidcyste
- ☐ Kolloidcyste

Selläre Tumore
- ☐ Hypophysenadenom
- ☐ Kraniopharyngeom

- ☐ Andere

Komplikationen

Gab es Komplikationen im peri-/postoperativen Verlauf? ○ ja ○ nein ○ unbekannt

Bildgebung und Diagnose

**Zerebrovaskulär**	Datum der aktuellen Bildgebung:			
	Aktuelle Bildgebung vorhanden?	○ Ja	○ Nein	○ unbekannt
**Aneurysma**	Komplette Occlusion/Resektion	○ Ja	○ Nein	○ unbekannt
	Bypass durchgängig	○ Ja	○ Nein	○ unbekannt
**AVM/dAVF/Cavernom**	Komplette Occlusion/Resektion	○ Ja	○ Nein	○ unbekannt
**Stroke**	Neue Ischämie	○ Ja	○ Nein	○ unbekannt
	Bypass durchgängig	○ Ja	○ Nein	○ unbekannt
	Wiedereröffnung Gefäss	○ Ja	○ Nein	○ unbekannt
**Tumor**	Datum der aktuellen Bildgebung:			
	Postop. CT/MRI vorhanden?	○ Ja	○ Nein	○ unbekannt
	In Tumorboard besprochen?	○ Ja	○ Nein	○ unbekannt

Tumorentfernung retrospektiv (anhand postop. CT/MRI)

Gliom

○ Complete resection ○ GTR(>98%) ○ Subtotal resection (>90%) ○ Partial resection (<90%) ○ Biopsy

Metastase / anderer Tumor

○ Totalentfernung ○ Partielle Entfernung ○ Biopsie

Austrittsstatus

Karnofsky-Index		
Modified Rankin Scale		
Modifizierte NIH Stroke Scale		
Verhalten	○ adäquat ○ auffällig ○ unbekannt	
Montreal Cognitive Assessment	max. 30 Punkte	☐ Nicht erhoben
Epileptischer Anfall postoperativ?	○ ja ○ nein ○ unbekannt	

4 Follow-up Case Report Form (fCRF)

CRF-Sprechstunde

Zustand derzeit

Zustand derzeit (AQC)
- ○ geheilt (erwartetes Resultat erreicht)
- ○ gebessert
- ○ nicht gebessert / unverändert
- ○ verschlechtert
- ○ nicht beurteilbar

Aufenthalt derzeit (PRISMA)
- ○ Todesfall
- ○ Alltagsumfeld (Privathaushalt o. Ä.)
- ○ Krankenheim, Pflegeheim
- ○ Altersheim, sozialmedizin. Institution
- ○ Psychiatrische Klinik
- ○ Rehabilitationsklinik
- ○ anderes Krankenhaus
- ○ andere
- ○ unbekannt
- ○ eigenes Spital / andere Abteilung

Behandlung derzeit (PRISMA)
- ○ Todesfall
- ○ geheilt oder kein Behandlungsbedarf
- ○ ambulante Behandlung
- ○ ambulante Pflege / Spitex
- ○ stationäre Behandlung
- ○ Rehabilitation
- ○ andere
- ○ unbekannt

Exitus Datum [ ]

Bildgebung und Diagnose

Tumor
Datum der aktuellen Bildgebung: [ ]
Aktuelle Bildgebung vorhanden? ○ Ja ○ Nein

Zerebrovaskulär
Datum der aktuellen Bildgebung: [ ]
Aktuelle Bildgebung vorhanden? ○ Ja ○ Nein

Komplikationen

Gab es Komplikationen im peri-/postoperativen Verlauf? ○ ja ○ nein ○ unbekannt

Soziales Umfeld und Ressourcen (Fragen an den Patienten)

Wie ist Ihre Gesundheit im Allgemeinen?
- ○ Sehr gut
- ○ gut
- ○ mittelmässig
- ○ schlecht
- ○ sehr schlecht

Sind Sie mit Ihrer Narbe zufrieden?
- ○ Ja
- ○ Nein

Rauchen Sie?
- ○ ja
- ○ nein, noch nie
- ○ nein, sistiert

Berufliche Situation derzeit
- ○ erwerbstätig 100% (auch wenn arbeitsunfähig / krank)
- ○ erwerbstätig Teilzeit (auch wenn arbeitsunfähig / krank)
- ○ in Ausbildung / Umschulung
- ○ Haushalt / Freiwilligentätigkeit
- ○ keine Tätigkeit
- ○ arbeitslos gemeldet
- ○ IV-Bezüger (Invalidenrente)
- ○ AHV-Bezüger (Altersrente)
- ○ andere
- ○ unbekannt

Karnofsky-Index [ ]

Modified Rankin Scale [ ]

Modifizierte NIH Stroke Scale [ ]

Verhalten ○ adäquat ○ auffällig ○ unbekannt

Montreal Cognitive Assessment [ ] max. 30 Punkte ☐ Nicht erhoben

Epileptischer Anfall seit letzter Visite? ○ ja ○ nein ○ unbekannt
